# Neutrophil Extracellular Traps in Tumor Metastasis: Pathological Functions and Clinical Applications

**DOI:** 10.3390/cancers13112832

**Published:** 2021-06-06

**Authors:** Qian Chen, Lu Zhang, Xiang Li, Wei Zhuo

**Affiliations:** 1 Department of Cell Biology and Gastroenterology, Sir Run Run Shaw Hospital, Zhejiang University School of Medicine, Zhejiang University, Hangzhou 310058, China; 12018078@zju.edu.cn (Q.C.); 21818639@zju.edu.cn (L.Z.); 21918007@zju.edu.cn (X.L.); 2 Cancer Center, Zhejiang University, Hangzhou 310058, China; 3 Institute of Gastroenterology, Zhejiang University, Hangzhou 310016, China

**Keywords:** neutrophil, neutrophil extracellular trap, tumor metastasis

## Abstract

**Simple Summary:**

Tumor-associated neutrophils constitute an important portion of the infiltrating immune cells in the tumor microenvironment. One of the abilities of neutrophils is forming neutrophil extracellular traps. Recent studies on tumor-associated neutrophils have drawn increasing attention to the role of neutrophil extracellular traps in the tumor microenvironment. There were also some reviews summarize the pro-tumorigenic activity of NETs in tumors. The specific novelty of this article is the specific summarization on the pivotal roles of NETs in tumor invasion-metastasis cascade and the recapitulation on the potential of NETs in clinical applications.

**Abstract:**

Neutrophil extracellular trap (NET) formation is an ability of neutrophils to capture and kill pathogens by releasing chromatin scaffolds, along with associated cytotoxic enzymes and proteases, into the extracellular space. NETs are usually stimulated by pathogenic microorganisms and their products, surgical pressure or hypoxia. Interestingly, a number of recent studies suggest that tumor cells can induce NET formation, which in turn confers tumor cell malignancy. Notably, emerging studies indicate that NETs are involved in enhancing local invasion, increasing vascular permeability and facilitating immune escape and colonization, thus promoting tumor metastasis. In this article, we review the pivotal roles of NETs in the tumor metastasis cascade. We also recapitulate the potential of NETs as a cancer prognostic biomarker and therapeutic target.

## 1. Introduction

Neutrophils are abundant and heterogeneous leukocytes in mammals that mediate against infection or injury. As the first responders, neutrophils are the initial host defense against harmful microorganisms, including bacteria, fungi and protozoa, primarily through phagocytosis and degranulation. In 2004, Brinkmann et al. first discovered a novel function of neutrophils called neutrophil extracellular traps (NETs). NETs are compounded from filamentous DNA scaffolds, histones and granular proteins [1]. The scaffold of chromatin immobilizes the invaders and provides a microenvironment that can bring pathogens close to antimicrobial peptides, while the antimicrobial compounds (such as antimicrobial peptides, histones and proteases) equipped in NETs can kill pathogens. They are therefore considered able to play a key role in host defense [2].

A large number of studies focused on NETs have already extended into other fields. It was demonstrated that excessive NET formation and/or reduced NET removal may promote infectious inflammation [3,4] and noninfectious inflammation, including diabetes and atherosclerosis [5]. A crucial part of the influence of NETs on inflammation was attributed to the modulation of cytokine and chemokine activity via NET-related proteases [6]. In addition, NETs have been found to be important in thrombogenesis [7,8] and autoimmune diseases such as rheumatoid arthritis, systemic lupus erythematosus and anti-neutrophil cytoplasmic antibody-associated vasculitis [9]. Tumor-associated neutrophils (TANs) constitute an important portion of the infiltrating immune cells in the tumor microenvironment. A high intratumor neutrophil density is correlated with metastasis at lymph node sites, tumor grade and the tumor stage axis [10]. The tumor and tumor microenvironment have been shown to control neutrophil recruitment, and TANs have been found to regulate tumor progression and control tumor growth [10]. Many patients with advanced cancer show a high level of neutrophilia. TANs are connected to a dismal prognosis, and the neutrophil-to-lymphocyte ratio has been introduced as a significant prognostic factor for survival in many cancer types, such as multiple myeloma, mesothelioma and pancreatic cancer [11,12,13]. These recent studies on TANs have drawn increasing attention to the role of NETs in the tumor microenvironment. In this article, we list aspects of the function and mechanisms of NETs in specific steps of the tumor metastasis cascade in particular, including degrading the ECM, disrupting blood vessel integrity, promoting thrombosis, facilitating immune escape, trapping tumor cells in capillaries and promoting their extravasation, predicting metastatic organotropism, promoting the proliferation of micrometastases and activating dormant cancer cells. We also recapitulate the potential of NETs and their components to act as cancer prognostic biomarkers and therapeutic targets.

## 2. NET Formation

### 2.1. Conventional Mechanism

NETotic cell death [14], unlike other cell death subroutines such as apoptosis, necrosis and pyroptosis, is a specific cytolytic death mechanism in which neutrophils form NETs by releasing chromatin scaffolds, along with associated cytotoxic enzymes and proteases, into the extracellular space. A large number of stimuli including pathogenic microorganisms [15] and their derivatives [16], physicochemical stimulation [7,17,18,19,20,21,22], inflammatory cytokines [18,23,24,25,26] and metabolites [27,28,29,30,31,32] have been shown to induce NETs (Table 1). The mechanism behind NET formation depends on the properties of the stimulation [3,33]. Stimuli such as PMA, LPS and various types of bacteria result in reactive oxygen species (ROS) production through activation of NADPH oxidase via the Raf-MEK-ERK pathway [34,35]. Neutrophils with excessive cytoplasmic ROS are more prone to form NETs [36]. Many other stimuli such as calcium ionophores, nigericin, certain microbes, UV light and some crystals lead to NET formation without NADPH oxide but requiring mitochondrial reactive oxygen species (mROS) [37]. ROS appear to be essential for NET formation, whether production is mediated by NADPH oxidase or mitochondria. These ROS productions allow the release of neutrophil elastase (NE) and myeloperoxidase (MPO) from neutrophil granules. NE translocates to the nucleus, where it partially degrades specific histones, promoting chromatin decondensation. Subsequently, MPO synergizes with NE in driving chromatin decondensation independent of its enzymatic activity [38,39]. On the other hand, several stimuli lead to the NLRP3 inflammasome pathway, potentially causing caspase-1 activation, while intracellular LPS and Gram-negative bacteria activate caspase-11. NE and caspases-1 and -11 cleave/activate gasdermin D (GSDMD) that forms pores in both nuclear and plasma membranes [38]. Alkaline pH and subsequent calcium influx lead to enzyme peptidylarginine deiminase 4 (PAD4) activation [22]. Histone citrullination by PAD4 leads to chromatin decondensation and can be detected as a biomarker of ongoing NET formation [22]. They all lead to a common outcome: the decondensation of chromatin, rupture of the nuclear membrane and cell membrane and, eventually, the extrusion of NETs. This rather lengthy process of NET formation supports neutrophils in continuing their antimicrobial battle even beyond their life span [34].

Sometimes, NETs are not necessarily released from the nucleus. Neutrophils can eject their mitochondrial DNA into the extracellular space in conditions requiring ROS [23]. This process is not associated with cell death and also does not limit the life span of these cells [23]. These results assigned a novel role for mitochondria in neutrophils to not only serve as an mROS generator but also as a NET DNA provider in the process of NET formation. In addition, it is worth noting the original function of mitochondria for ATP production. Optic atrophy 1 (OPA1) is a mitochondrial inner membrane protein. Amini et al. showed that OPA1-dependent ATP production in neutrophils is required for NET formation [40]. A lack of OPA1 caused mitochondrial dysfunction and caused neutrophils to lose their ability to form functional NETs [40]. Up to now, the specific process of NETs derived from mitochondrial DNA is still unclear because mitochondrial DNA is very rare in neutrophils compared with nuclear DNA, and whether this difference reflects the need for adapting distinct physiological statuses or plays any role needs to be investigated further.

Moreover, neutrophils do not need to pay the price of cell death or cytolysis to form NETs [6]. They can squeeze out nuclear DNA through vesicle transport mechanisms [17]. This process is uniquely rapid (5–60 min) and does not involve ROS or NADPH oxidase but requires strict regulation mediated by TLR2 and a complement [26]. Pilsczek et al. observed separation of the inner and outer nuclear membranes and budding of vesicles, and the separated membranes and vesicles were filled with nuclear DNA. The vesicles were extruded intact into the extracellular space where they ruptured, which was followed by the release of chromatin [17]. However, how these large vacuoles are released remains unclear. Neutrophils without nuclei, called “cytoplasts”, have intact cell membranes and retain physiological characteristics such as phagocytosis [17]. NETs and cytoplasts have recently been found in the lungs and lymph nodes of asthmatic mice and in bronchoalveolar fluid collected from patients with severe asthma [41]. These cytoplasts are associated with activation of dendritic cells to differentiate naive CD4+ T cells into helper T17 effector cells [41], suggesting that after NET formation, the formed cytoplasts can satisfy the needs of normal biological functions.

In general, the main mechanisms of NET formation have already been well studied, but there are numerous interesting scientific problems in the intracellular process of NET formation waiting to be formulated and replenished.

### 2.2. Tumors Induce NET Formation

An increasing number of studies have revealed that tumor cells and the tumor microenvironment can stimulate neutrophils and induce NET release in various cancer types, including leukemia [48], breast cancer [48,64], ovarian cancer [65], colon cancer [66], esophageal gastric adenocarcinoma [66] and lung cancer [48,66].

**Figure 1:**
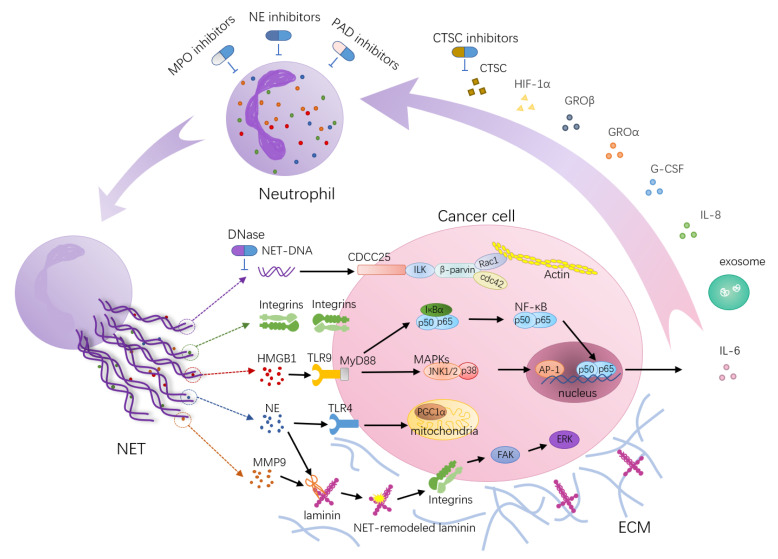
Neutrophil extracellular trap (NET)-associated molecular mechanisms’ function in tumor metastasis. NETs act as scaffolds mediating the capture of cancer cells and providing a microenvironment that can bring protumorigenic proteins close to cancer cells. HMGB1, a highly conserved DNA binding protein, is one of the components of NETs. NETs trigger HMGB1 release and activate TLR9-dependent pathways in cancer cells. The tumorigenic effects of TLR9 depend on NF-κB-mediated upregulation of IL-6 expression and activation of a cascade of intracellular growth signaling pathways, including MAP kinase pathways. NE released from NETs activates TLR-4 on cancer cells, leading to PGC-1α upregulation, increased mitochondrial biogenesis and accelerated growth. NETs trap circulating tumor cells via β1-integrin-mediated interactions. NE and MMP-9 sequentially cut laminin, an important component of the ECM, revealing an epitope that triggers the proliferation of dormant cancer cells through integrin activation and FAK/ERK/MLCK/YAP signaling. Furthermore, the transmembrane protein CCDC25 is a NET DNA receptor on cancer cells that senses extracellular DNA and subsequently activates the ILK-β-parvin pathway to enhance cell motility. In turn, certain factors secreted by many primary tumors have been shown to promote NET formation, such as cytokines (HIF-1α, IL-8, G-CSF, GROα, GROβ), proteases (CTSCs) and exosomes. During these processes, targets for therapies have been postulated, and interfering drugs (blue arrows) have already been used in clinical practice or are under investigation in vivo.

The formation of NETs may be partly due to the hypoxic environment in which growing solid tumors are generated concomitant with the higher expression of HIF-1α [20,21]. Moreover, secreted cytokines [18,48,58,64,67], proteases [68] and even exosomes [57] may also contribute to NET formation (Figure 1). Depletion of IL-8, G-CSF, GROα or GROβ derived from ovarian cancer cells can incompletely reduce NET formation and neutrophil chemotaxis, suggesting that these cytokines cooperate with each other to optimally stimulate neutrophil mobilization and NET formation [65]. Xiao et al. found that the tumor-secreted protease cathepsin C (CTSC) enzymatically activates neutrophil membrane-bound proteinase 3 (PR3) to upregulate IL-6 and CCL3 for neutrophil recruitment. In addition, the CTSC–PR3–IL-1β axis induces the formation of NETs which support the metastasis of cancer cells in the lungs [68]. Furthermore, tumor-derived exosomes have been closely linked to NET formation. Leal et al. found that tumor-derived exosomes of cancer patients with prethrombotic states can induce NET release, and that NETs can serve as a scaffold for tumor-derived exosomes and recruit them [58]. Colorectal cancer cells can transfer mutant KRAS to neutrophils through exosomes, thereby promoting NET formation by mediating upregulation of IL-8, ultimately leading to colorectal cancer deterioration [57]. This evidence supports the hypothesis that tumors can effectively induce NET formation by modulating the tumor microenvironment.

## 3. NETs Promote Tumor Metastasis

Metastasis is the major cause of cancer-related mortality. Tumor metastasis is a process in which tumor cells leave the primary site and reach distant tissues or organs where they form secondary lesions. Metastasis involves a series of events [69]: (1) local invasion, which is facilitated by breakdown of the extracellular matrix (ECM); at the same time, the release of cytokines embedded in the ECM further promotes the growth and survival of tumor cells; (2) intravasation into the tumor vasculature; (3) escape of circulating tumor cells from the immune system; (4) arrest in capillaries at the distant site and extravasation into the parenchyma of target organs; (5) entry into dormant tumor cells and reactivation; and (6) colonization and development of macrometastases.

**Figure 2:**
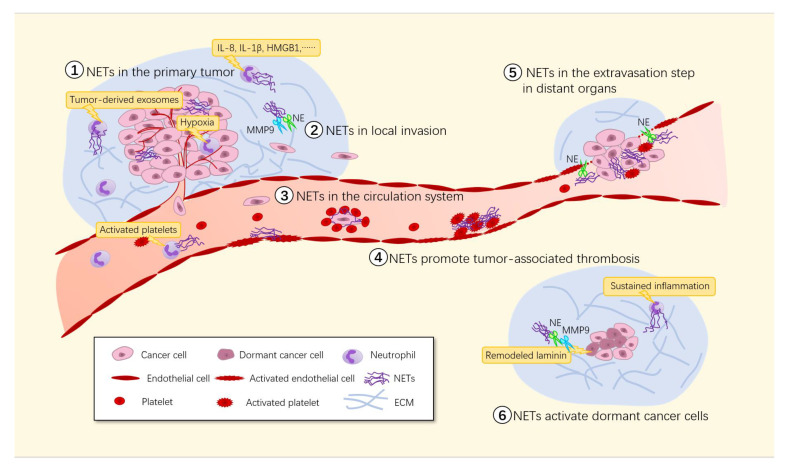
Involvement of NETs in tumor metastasis. Many stimuli including the hypoxic environment in growing solid tumors, inflammatory cytokines and activated platelets trigger NET formation in the tumor microenvironment. ① The existence of NETs promotes primary tumor growth through both the direct effect of NET components on the tumor cells themselves and the indirect effect of NETs on other components of the tumor microenvironment. ② Proteases in NETs can degrade the ECM, which subsequently releases cytokines to promote tumor cell growth and survival. ③ NETs can cover CTCs with platelets, creating a physical barrier between immune cells and CTCs that is difficult to penetrate. ④ Adhesion of massive NETs to the vasculature may initiate thrombosis by providing a scaffold for platelet adhesion, activation and thrombin generation. ⑤ NETs capture tumor cells and bind them to vascular walls via von Willebrand factor (VWF), disrupting the normal connections between endothelial cells and increasing the permeability of blood vessels, making it easier for tumor cells to break through the vascular walls to reach distant organs and form micrometastases. ⑥ The NET-associated proteases NE and MMP-9 can activate integrin α3β1 signaling by remodeling laminin, thus inducing the proliferation of dormant cancer cells.

Recently, emerging studies have demonstrated that NETs participate in the entire invasion–metastasis cascade process. In mouse models of lung cancer [62], ovarian cancer [65], colorectal cancer [21], pancreatic cancer [70] and breast cancer [71], depletion or inhibition of NET formation significantly reduced the number of tumor metastases. Here, we evaluate the role of NETs in tumor metastasis (Figure 2).

### 3.1. NETs in the Primary Tumor

#### NETs Promote Tumor Growth

It has been reported that the existence of NETs promotes the growth of many types of tumors. In chronic lymphocytic leukemia, it was demonstrated that NETs directly induce CD5+ B cell proliferation by activating the NF-κB signaling pathway, providing a proof of concept that NETs may directly influence tumor cell growth [54]. Neutrophils contain relatively few mitochondria and derive most of their energy from glycolysis [72]. NE released from NETs can increase mitochondrial biogenesis through PGC-1 upregulation via activation of TLR-4 in cancer cells, further accelerating colorectal tumor growth [73]. In pancreatic tumors, DNA from neutrophils activates pancreatic stellate cells that form dense and fibrous stroma, which can initiate and promote tumor proliferation [70]. In addition, Demers et al. found the presence of NETs increased Lewis lung carcinoma tumors and melanotic tumor growth [24].

These studies showed that NETs lead to a growth advantage for tumors not only by directly proliferating tumor cells but also by indirectly altering the tumor microenvironment and altering tumor metabolism.

### 3.2. NETs in Local Invasion

#### NETs Degrade the ECM

Degradation of the ECM to break through the basement membrane is the first step in tumor cell invasion and metastasis. The NET DNA backbone can act as a proteolysis scaffold, ornamented by a variety of proteases such as NE, matrix metalloproteinase 9 (MMP9) and cathepsin G (CG).

NE can be released from neutrophils independent of NET formation but can be rapidly inactivated by plasma antiproteases [74]. Belorgey et al. indirectly proved that NE activity is still present in NETs because the DNA retains the proteolytic activity of NE for extended periods by suppressing the effect of the anti-elastase control system [74,75]. It was also proved that NE plays an important role in the pathological functions of NETs. Recent research revealed that released NE and MMP-9 in NETs can sequentially cleave laminin, which is an important component of the ECM [19]. However, there are a few direct pieces of evidence about NE’s and other proteases’ activity in NETs. The majority of studies only analyzed the role of neutrophil-related granule proteins in the process of NET formation but did not pay attention to these enzymes’ activity after the release of these proteins from neutrophil granules. The functions of neutrophil-related granule proteins in NETs and the initiation process of tumor metastasis still need further exploration.

### 3.3. NETs in Vascular Permeability

#### 3.3.1. NETs Disrupt Blood Vessel Integrity

The maintenance and stability of vascular integrity primarily depend on the tight connection between vascular endothelial cells. NETs have been reported to increase vascular permeability to promote metastasis.

NETs that persist in the microcirculation can stimulate vascular endothelial cells to endocytose NETs. Intracellular NET-associated elastase can promote nuclear translocation of junctional β-catenin and lead to downregulation of the intercellular connection protein VE-cadherin which further induces endothelial-to-mesenchymal transition [76]. However, endothelial cells have a limited capacity to internalize NETs via the receptor of advanced glycation end-products. An overflow of the phagocytic capacity of endothelial cells for NETs resulted in the persistent extracellular presence of NETs, which rapidly altered endothelial cell–cell connections and induced transendothelial albumin leakage through elastase-mediated proteolysis of the intercellular junction protein VE-cadherin [76]. In vivo animal experiments have shown that NETs capture tumor cells and bind them to vascular walls via von Willebrand factor (VWF) [77]. NETs possess NE proteolytic activity to disrupt the tight connection between endothelial cells and increase the permeability of blood vessels [77].

Similar phenomena were also observed in autoimmune disorders (such as lupus nephritis) [76] and infectious inflammation (such as bloodstream infection with methicillin-resistant *Staphylococcus aureus*) [77]. The underlying mechanisms of NET-mediated interactions between tumor cells and endothelial cells deserve further research.

#### 3.3.2. NETs Promote Thrombosis

Cancer-associated thrombosis is linked to poor prognosis and is the second leading cause of death in cancer patients but often lacks a clear etiology [78]. Nucleic acids and nuclear components have been shown to induce coagulation [79]. Recent studies have shown that excessive NET release in tumor tissue is procoagulant and prothrombotic. Thalin et al. found that the cancer-induced systemic NET burden resulted in widespread arterial thrombotic events in the brain and heart injury [80]. The relationship between NETs and cancer-associated thrombosis has also been found in other cancer types. In the late stages of a breast carcinoma model, NET formation occurs concomitantly with the appearance of venous thrombi in the lung [48]. Moreover, Wolach et al. found that neutrophils from patients with myeloproliferative neoplasms (MPNs), characterized by a mutation constitutively activating JAK2 signaling, were also primed for NET formation. Inhibition of constitutively active JAK2 abrogated NET formation and reduced thrombotic events associated with the mutation [36], suggesting the importance of neutrophils and NETs in cancer-associated thrombosis. NET-associated microthrombi and high circulating levels of G-CSF were also detected in patients with ischemic stroke and underlying cancer, further linking a cancer-induced systemic NET burden to widespread arterial microthrombosis [80].

NETs promote thrombosis in several ways. Fuchs et al. demonstrated that NETs provide a physical scaffold for thrombus growth by capturing platelets and red blood cells [7]. Platelets interact with NETs through DNA–histone complexes or by binding to plasma proteins, such as VWF and fibronectin [7]. In addition, NETs can activate platelets and coagulation factors to stimulate coagulation [38]. Moreover, tumor-derived exosomes, found in cancer patients with a prothrombotic state, have been shown to stimulate NET formation [58], bind to NETs [80] and cooperatively accelerate venous thrombosis in tumor-free mice [58,80].

These studies provide a new insight into the role of NETs in inducing thrombosis. It will be interesting to explore whether NET-induced thrombosis is involved in tumor metastasis. NET inhibitors may reduce cancer-associated thrombosis and improve the prognosis of cancer patients.

### 3.4. NETs in the Circulation System

#### NETs Promote Immune Escape

Circulating tumor cells (CTCs) are cancer cells that circulate in the bloodstream after being naturally shed from the original or metastatic tumor and may lead to a new metastasis [81,82]. Millions of tumor cells are released into the circulation system every day, yet many cancer patients never relapse after a long period of latency without clinically manifesting disease. This might be because the CTCs do not survive in the blood circulation during the process of metastasis, and only a minority of the surviving CTCs can successfully establish new metastatic clones [69].

CTCs must survive blood flow shear forces and immune system challenges [83]. Based on the trapping characteristics of NETs, some reviews have speculated that NETs can cover CTCs with platelets, creating a physical barrier between immune cells and CTCs that is difficult to penetrate [84,85]. However, evidence to support this speculation is scant. Very recently, these conjectures were verified in succession [86,87]. Ren et al. found that surgical stress-activated platelets enhance the formation of platelet–tumor cell aggregates, facilitating their entrapment by NETs and subsequent distant metastasis. A murine hepatic ischemia/reperfusion (I/R) injury model of localized surgical stress showed that depletion of platelets inhibited the capture of CTCs by NETs and eventual metastasis to the lungs. Blocking platelet activation or knocking out TLR4 protected mice from hepatic I/R-induced metastasis with no CTC entrapment by NETs [86]. Teijeira et al. observed that NETs wrap and coat tumor cells in intravital microscopy. In their hands, NETs shield tumor cells from cytotoxicity, as mediated by CD8+ T cells and natural killer cells, by obstructing contact between immune cells and the surrounding target cells [87].

These results reveal a novel detail of the mechanism of tumor immune escape. Targeted disruption of the interaction between platelets, tumor cells and NETs holds therapeutic promise to prevent postoperative distant metastasis.

### 3.5. NETs in the Extravasation Step in Distant Organs

#### NETs Trapping Tumor Cells in Capillaries and Promoting Their Extravasation

Once CTCs are entrapped in or adhered to capillaries, they extravasate into the surrounding tissue and form micrometastases [88]. This extravasation is crucial for metastasis [88]. NETs can increase the sequestration of tumor cells in distant organs by trapping tumor cells in capillaries, which leads to increased colonization.

Cools-Lartigue et al. reported extensive NET deposition in the microvasculature of peripheral organs in systemic sepsis, resulting in increased tumor cell adhesion to the hepatic and pulmonary microvasculature in vivo [62]. Images in this study obtained via confocal or electron microscopy demonstrated cancer cells trapped within webs of extracellular DNA, while neutrophils could not directly contact tumor cells, suggesting that the adhesive mechanism was mainly mediated by trapping within NETs [62]. Using preclinical murine models of lung and colon cancer in combination with intravital video microscopy, Rayes et al. confirmed that NETs promote adhesion of CTCs to the lung and liver, thereby functionally promoting metastasis progression. Blocking NET formation through multiple strategies significantly inhibits spontaneous metastasis [66]. Najmeh et al. demonstrated that β1-integrin is an important factor mediating the interactions between CTCs and NETs. They showed that β1-integrin expression on both cancer cells and NETs is important for the adhesion of CTCs to NETs both in vitro and in vivo. The early events of extravasation can be eliminated by blocking NETs using various strategies [89]. To intervene in this process, further research is needed to understand the exact molecular nature of the underlying mechanisms.

### 3.6. NETs in Organotropic Metastasis

#### NETs Predict Metastatic Organotropism

There is abundant evidence that certain cancer cells have a highly organotropic preference to colonize certain distant organs and to establish metastases. Ovarian cancer cells usually preferentially seed in the lungs, liver, bones and omentum [90]. It is easier for colon cancer cells to metastasize to the liver and lung [91]. The organ-specific homes for metastases are considered premetastatic niches, predetermined microenvironments induced by tumors in distant organs, that are conducive to the survival and outgrowth of tumor cells before their arrival [92].

Neutrophils have been linked to the formation of the premetastatic niches. Lee et al. found that early-stage ovarian tumors, especially those of high metastatic potential, induce NET formation in the premetastatic omental niche. Subsequent studies showed that NET formation in the premetastatic omental niche promotes the implantation of ovarian cancer cells. Moreover, ovarian cancer cells showed reduced metastasis to the omentum in NET-deficient mice, suggesting the possibility that blockade of NET formation can prevent omental metastasis [65]. Surprisingly, rather than merely acting as a “trap” for “passer-by” cancer cells, NET DNA also acts as a chemotactic factor to attract cancer cells. Yang et al. identified the transmembrane protein CCDC25 as a NET DNA receptor of cancer cells that senses extracellular DNA and subsequently activates the ILK-β-parvin pathway to enhance cell motility. They found that NETs are abundant in the liver metastases of patients with breast and colon cancers, which generally correspond to metastatic organotropism of these cancers [93]. A recent study reported further mechanisms of interaction between NETs and tumor cells in lung premetastatic niches of breast cancer. Xiao et al. found that the tumor-secreted protease cathepsin C (CTSC) promotes breast-to-lung metastasis by regulating the recruitment of neutrophils and formation of NETs [68].

Overall, these findings indicate that the level of NETs formed in distant organs reflects the metastasis potential and organotropism of the tumor and that detection of NETs can be used for treatment selection and prognosis estimation.

### 3.7. NETs in Micrometastases/Colony Formation

#### 3.7.1. NETs Promote the Proliferation of Micrometastases

Many patients already have tiny tumor deposits at the time of surgery that may contribute to postoperative tumor recurrence [94]. Cools-Lartigue et al. proposed that following sequestration within NETs, CTCs are able to form stable micrometastatic foci and ultimately grow to form macrometastases [95]. This implies that trapped cancer cells can not only survive but also grow and proliferate. This phenomenon was also confirmed in other studies. A time-lapse video revealed that human gastric cancer cells trapped by NETs did not die but grew vigorously in continuous culture [96]. Tohme et al. demonstrated that NETs affect the growth of existing micrometastases in animal models. Mice treated with DNase displayed significantly decreased tumor growth with smaller and less numerous tumors [21]. After abdominal surgery, NETs on the peritoneum gather the disseminated tumor cells and provide a favorable microenvironment for the survival of the cells [96].

These results support the hypothesis that NET formation plays an important role in micrometastasis growth, and disruption of NETs may be clinically useful to prevent postoperative tumor recurrence.

#### 3.7.2. NETs Activate Dormant Cancer Cells

Cancer cells that have disseminated to distant tissues are able to remain dormant for years, even decades, before relapsing or awaking. T cells and natural killer cells can eliminate disseminated cancer cells as they begin to proliferate, which keeps cancer cells at a clinically undetectable level [97,98,99]. The mechanisms by which dormant cancer cells become awakened or resume proliferation remain largely unknown.

Several recent studies found that the formation of NETs can activate dormant tumor cells [19]. Albrengues et al. found that sustained lung inflammation caused by tobacco smoke exposure or nasal instillation of lipopolysaccharide converted disseminated, dormant cancer cells to aggressively growing metastases [19] in mouse models. Mechanistic analysis revealed that two NET-associated proteases, NE and MMP-9, sequentially cleaved laminin-411 and laminin-511 in the niche around blood vessels, which has been shown to regulate the dormant state of breast cancer [100]. Proteolytically remodeled laminin induces proliferation of dormant cancer cells by activating integrin α3β1 signaling [19].

Therefore, the postoperative lifestyle of cancer patients is significant for preventing tumor recurrence. Inhibitors against NETs may prevent dormant cells from recovering and extend the survival time of cancer patients.

## 4. The Clinical Significance of NETs

Recent studies have shown that NETs are closely related to tumor metastasis and have significant clinical relevance. Patients with advanced cancer had higher levels of circulating NETs than healthy individuals and even local cancer patients [66,70]. In addition, high levels of NETs were associated with a worse prognosis. High circulating levels of NETs in colorectal cancer were associated with more postoperative complications and higher cancer recurrence rates [21,85,101]. Tumor-infiltrated NETs in pancreatic ductal adenocarcinoma predict poor postoperative survival [102]. In Ewing sarcoma, patients with high circulating levels of NETs and NET deposition in tumor tissue samples were prone to metastasis and early recurrence after intensive chemotherapy [103,104]. Yang et al. found that the level of serum NETs predicted the occurrence of liver metastasis in patients with early-stage breast cancer [93]. These clinical results suggest the potential of NETs as a biomarker for diagnosis and prognosis as well as a target for therapy and interference.

### 4.1. NETs as a Cancer Biomarker

As a cancer biomarker, NET levels are usually detected in tumor tissues or serum. Diverse NET-associated molecules can be conveniently detected such as citrullinated histone H3 (citH3) [105,106,107], MPO [21,64], NE [64,108], HMGB1 [52,53], histone H1 [64], histone H2 [16] and histone H4 [25]. Other putative biomarkers of NETs include cell-free DNA (cfDNA) and nucleosomes, which may sometimes be contaminated by cellular decay or apoptosis [106]. In addition, SytoxGreen, a cell-impermeable dye, can be used to stain extracellular DNA because it intercalates into cell-free DNA and enters dead cells but not live cells [65]. CitH3 is formed as a result of PAD4-mediated citrullination during NET formation and is the most specific biomarker of NETs [107]. Higher levels of citH3 were observed in the plasma of patients with advanced malignancies than in healthy individuals or patients without cancer [105]. In fact, elevated circulating citH3 levels have been associated with high mortality in cancer patients [107]. In addition, based on the report that citH3 levels in plasma can predict the risk of venous thromboembolism in cancer patients [106], several clinical trials are currently preparing to quantify NET levels as a tumor associated-thrombosis biomarkers in myeloproliferative neoplasms (NCT04177576, https://clinicaltrials.gov, accessed on 26 November 2019), pancreatic cancer, gastric cancer and colon cancer (NCT04294589, https://clinicaltrials.gov, accessed on 4 March 2020). Similarly, according to MPO-DNA quantification, NET-specific DNA was significantly correlated with the clinical stage of pancreatic cancer at presentation [70]. Metastatic colorectal cancer patients showed increased intratumoral NETs in tissues and aberrant levels of preoperative serum MPO-dsDNA. Higher MPO-dsDNA levels were correlated with a shorter survival time [73]. These studies suggest NETs as biomarker candidates to guide clinical diagnosis and treatment and to assess the prognosis of cancer patients. However, we should be cautious about the standard of “normal” levels of NETs, since NET levels may be aberrant in patients with other diseases [43].

### 4.2. NETs as a Therapeutic Target

Considering the significant prometastatic functions of NETs, targeting NETs can be a promising approach against tumor metastasis. Animal studies have shown inhibition of tumor metastasis by blocking NET formation with the application of small molecule drugs such as DNase [21,58,62,66], CTSC inhibitors [68], PAD4 inhibitors [19,64,109,110] and NE inhibitors [62,66]. DNase I treatment suppressed the development of gross metastases and the growth of established liver micrometastases in metastatic colorectal cancer animal models [21]. AZD7986 is a second-generation CTSC inhibitor and a therapeutic candidate for neutrophil-driven inflammatory diseases, such as chronic obstructive pulmonary disease [111,112]. Interestingly, targeting CTSC with the compound AZD7986 effectively suppressed circulating pulmonary NETs and alleviated lung metastasis of breast cancer in a mouse model, but there was no effect on primary tumor growth [68]. The PAD4 inhibitor Cl-amidine significantly reduced NET formation, but the number of breast cancer cells that extravasated into the lung tissue was not altered [64]. Another PAD4 inhibitor, GSK484, was recently shown to prevent tumor-associated renal dysfunction in mice, and the effect was determined to be NET-mediated [109,110]. By preventing NET formation through DNase or NE inhibitors and in PAD4-defective mouse models, NET-deficient mice showed reduced spontaneous lung and liver metastasis of lung carcinoma cells [66].

The development of clinical therapies specifically targeting NETs in cancer is in its infancy. For instance, DNase has already been in clinical use for decades for the management of cystic fibrosis, demonstrating its safety as a drug [113]. Therefore, some clinical tests have evaluated the effectiveness of DNase in cancer. Pulmozyme, a recombinant human DNase, has been evaluated in a phase 1 trial in head and neck cancer patients treated with radiotherapy and chemotherapy (NCT00536952, https://clinicaltrials.gov, accessed on 28 September 2007). In a phase 2 clinical trial, combination treatment with Oshadi D (DNase in an Oshadi carrier) and Oshadi R (RNase in an Oshadi carrier) was shown to have antitumor activity and a good safety profile in patients with acute myeloid leukemia or acute lymphoid leukemia (NCT02462265, https://clinicaltrials.gov, accessed on 4 June 2015). In addition, metformin is a well know antidiabetic drug, which is used since many decades and its pharmacology is well characterized. Metformin is also widely used in cancer treatment, but its mechanism is not completely understood. A recent clinical trial revealed an interesting anti-NET activity of metformin, this effect was related to the inhibitory effect exerted by metformin on the PKC-NADPH oxidase pathway [114]. Moreover, hydroxychloroquine, an autophagy inhibitor, can inhibit NET formation [115]. Correlative data from patients with pancreatic adenocarcinoma suggested that treatment with hydroxychloroquine diminished hypercoagulability and reduced the perioperative venous thromboembolism rate from 30 to 9.1% [116]. However, the consequences of suppressing NET formation must be carefully evaluated. Injection of these NET inhibitors may have off-target effects, including compromising the immune function of NETs. It is possible that some patient groups, such as elderly cancer patients with compromised immunity, are not suited for this type of therapy.

These findings support the potential of NET-targeting approaches for cancer treatment. Therefore, the molecular mechanisms underlying the role of NETs in tumor metastasis should be further studied to supply referential data for clinical treatment.

## 5. Conclusions

In the tumor microenvironment, neutrophils have varied functions that influence cancer development and progression. Recent studies have shown that NETs, a novel function of neutrophils, play a vital role in tumor progression, which opens a new research vision of neutrophils in tumor metastasis.

In tumor progression, NETs participate in the overall invasion–metastasis cascade response, and the molecular mechanisms underlying the role of NETs in these processes need to be further studied to provide evidence for clinical treatment. In addition, like macrophages, TANs may acquire either an antitumor activity (N1 TANs) or protumor activity (N2 TANs) [10]. Whether different TAN subtypes have different NET formation abilities, which TAN subtype the NETs studied thus far have employed and whether NETs from different sources have different functions remain elusive. Moreover, some studies suggest that NETs not only directly influence tumor cells but also influence other cells in the tumor microenvironment, such as macrophages [30], vascular endothelial cells [76] and pancreatic stellate cells [70]. However, these studies involved only a few cell types, and a more comprehensive understanding of the role of NETs in the tumor microenvironment is needed.

For clinical studies, the relevance of NET levels to mortality, clinical stage and the survival time of cancer patients shows the potential of NETs to guide clinical diagnosis as a cancer biomarker candidate. Therefore, it is necessary to improve the NET detection method and ascertain a detection standard. It is difficult to identify which diseases the detected NETs are derived from, which is a challenge in utilizing NETs as a cancer biomarker. The detection of neutrophil cytoplasts has the potential to be a complementary method with NET detection, but there are few studies on neutrophil cytoplasts. Another major problem is the lack of standardization in NETs. It is difficult to integrate and evaluate NET levels in healthy people and patients because researchers evaluate NET levels in their own systems. To date, the drugs targeting NETs have a beneficial effect in animal models, and some NET inhibitors have been screened as drug candidates due to their safety and have shown potential for improving cancer treatment; however, in cancer patients, recent studies have not provided conclusive evidence of the efficacy of this approach. Indeed, it will be necessary to focus subsequent research on drug therapy targeting NETs. It has been reported that NETs have a direct therapeutic effect playing an immune regulatory role in bladder cancer [117,118]. Therefore, balancing between the immune function and the tumor-promoting action of NETs by regulating the dosage and delivery methods of drugs as much as possible is a significant challenge. Clinical trials of NET-targeted drugs in cancer patients can be carried out gradually. Targeting NETs might be a promising approach against tumor metastasis.

## Figures and Tables

**Table 1:** List of stimulants known to induce NET formation and the relation to tumor metastasis.

Stimulus		Potential Mechanisms to Induce NET Formation	The Role of NET in Tumor Progression	Reference
**Cytokines**	PAF	-	Promotes tumor cell proliferation, neovascularization and immunosuppressive phenotype	[42,43]
	IL-8	Activation of the class I isoform of PI3K	Positive correlation with poor outcome in women with breast cancer; enhances angiogenesis and contributes to tumor growth and progression	[18,44,45]
	IL-1β	Nuclear localization of ceramide synthase 6 and synthesis of C16-ceramide induce NETs	Promotes abdominal aortic aneurysm formation	[46,47]
	GM-CSF	-	Promotes tumor growth and metastasis	[23,24,48,49]
	CLL7	-	-	[25]
	Complement factor 5a (C5a)	-	-	[23,50]
	TNF-α	-	-	[51]
	High-mobility group box 1 protein (HMGB1)	Interactions between HMGB1 and neutrophil-derived TLR4	Activates TLR9-dependent pathways in cancer cells to promote tumor malignancy	[21,52,53]
	IFNs	Induce strong tyrosine phosphorylation of STAT1 in mature neutrophils	-	[50,54]
	HIF-1α	-	-	[20]
	P-selectin	Promotes NET formation through binding to anti-P-selectin glycoprotein ligand-1 (PSGL-1)	-	[42]
**Metabolite**	Urate crystals	Interact with lysosomes and result in secretion of IL-1β to induce NADPH oxidase-independent NET formation	-	[27,47]
	Lactic acid	-	-	[55,56]
	Free fatty acid	-	-	[29]
	Cholesterol crystal	-	-	[30]
	2-chlorofatty aldehyde and 2-chlorofatty acid	As an MPO product to trigger NET formation following neutrophil activation	-	[31]
	High glucose	-	-	[32]
	Tumor-derived exosomes	-	KRAS mutation in exosomes causes deterioration of colorectal cancer	[57,58]
	Immobilized immune complexes	Induce FcγRIIIb-mediated NADPH oxidase-independent NET formation	-	[59]
	Activated platelets	-	-	[60]
	Mitochondrial DNA	Triggers TLR9-dependent NET formation	-	[61]
**Physical** **stimulation**	Hypoxia	-	-	[20,21]
	Surgical stress	-	Accelerates development and progression of liver metastatic disease	[21,62]
	UV light	NADPH oxide-independent NET formation but requiring mROS	-	[37]
**Chemical stimulation**	PMA	Triggers assembly and activation of NADPH oxidase and ROS production via the Raf-MEK-ERK pathway	-	[1,34]
	Hydrogen peroxide (H_2_O_2_)	Stimulates activation of NADPH oxidase and the production of ROS	-	[34]
	LPS	Induces inflammation, triggers the assembly and activation of NADPH oxidase and the production of ROS via the Raf-MEK-ERK pathway and activates caspase-11 to activate gasdermin D	Activates dormant cancer cells and enhances metastatic proliferation	[16,19]
	fMLP	Activates PI3K and MAPK pathways	-	[18,19]
	Cigarette smoke extract	-	Converts dormant cancer cells to aggressively growing metastases	[19]
	Alkaline pH	Promotes intracellular calcium influx, mROS generation, PAD4-mediated CitH3 formation and histone 4 cleavage	-	[22]
	Tamoxifen	Modulates intracellular ceramide via a ceramide/PKCζ-mediated pathway	-	[63]
	Nitric oxide (NO)	-	-	[51]
	Calcium ionophores	NADPH oxidase-independent NET formation but require mROS	-	[22]

## Data Availability

Data sharing is not applicable to this article.
